# The Triglyceride–Glucose Index: A Clinical Tool to Quantify Insulin Resistance as a Metabolic Myocardial Remodeling Bridge in Atrial Fibrillation

**DOI:** 10.3390/biomedicines13102348

**Published:** 2025-09-25

**Authors:** Muhua Luo, Yaping Wang

**Affiliations:** 1 Department of Cardiology, The Second Affiliated Hospital, School of Medicine, Zhejiang University, Hangzhou 310009, China; 2 State Key Laboratory of Transvascular Implantation Devices, Hangzhou 310009, China; 3 Heart Regeneration and Repair Key Laboratory of Zhejiang Province, Hangzhou 310009, China

**Keywords:** triglyceride–glucose index, insulin resistance, atrial fibrillation

## Abstract

Atrial fibrillation (AF) is the most common arrhythmia worldwide, a major cause of heart failure and stroke, and carries a significant healthcare burden. Atrial cardiomyopathy (ACM) provides the structural and electrophysiological basis for AF, while metabolic dysregulation, particularly insulin resistance (IR), is increasingly recognized as a key factor exacerbating atrial remodeling. However, due to the complexity and high cost of IR measurement procedures, no reliable, user-friendly, and practical tool supporting AF risk stratification has yet been identified in clinical practice. To address this gap, we conducted a literature search in various databases and found an increasing body of research indicating that the triglyceride–glucose index (TyG index) is a simpler, more cost-effective alternative for identifying adverse metabolic profiles and the AF risk. This narrative review describes the existing literature and explores the potential mechanisms underlying changes in the TyG index and its clinical applications, while also discussing the challenges facing the use of this index and future research directions. In summary, the current evidence suggests that the TyG index is a promising but not yet established risk assessment biomarker for AF.

## 1. Introduction

Atrial fibrillation (AF) is the most common clinical arrhythmia, with its incidence and prevalence having risen over the past few years [1,2]. Characterized by the loss of regular atrial electrical activity, AF results in rapid and disordered atrial fluttering.

Currently, more than 30 million people worldwide suffer from AF [1,2]. The Global Burden of Disease (GBD) study revealed a continuous increase in the prevalence, incidence, and mortality rates of AF worldwide, with some regions in North America and Europe showing the highest prevalence rates and China, Southeast Asia, South Asia, the Middle East, and Africa exhibiting the lowest. This disparity is primarily attributable to differences in diet and lifestyle [3]. Furthermore, the AF detection methods used in these regions face limitations, meaning that the actual number of affected individuals exceeds that found by surveys [4,5,6]. Data from the Optum commercial insurance database in the United States shows that AF patients have a higher risk of hospitalization and cardiovascular complications (e.g., heart failure, stroke), which leads to increased healthcare costs and significant burdens on individuals and the healthcare system [7,8,9]. Therefore, developing effective strategies for the prevention, diagnosis, and management of AF is of the utmost importance.

With in-depth investigation of the mechanisms of atrial pathology, a key entry point for AF management has become the examination of atrial cardiomyopathy (ACM) as the core pathologic basis for the development of AF and a risk factor for stroke [10]. The 2024 European Heart Rhythm Association (EHRA) consensus states that ACM encompasses structural, electrophysiological, inflammatory, and metabolic abnormalities and can be subdivided into four types, each with distinct features: type 1, myocyte degeneration associated with impaired energy metabolism; type 2, fibrosis involving fibroblast proliferation, cardiomyocyte hypertrophy, and excessive extracellular matrix deposition that disrupts the conduction continuity; type 3, fatty infiltration or hypertrophy that alters the electrical conduction substrate; and type 4, fascicular disarray that directly leads to conduction anisotropy [11].

Notably, metabolic dysregulation plays a critical pathophysiological role in ACM, particularly within type 1 and type 3 [12,13]. This dysfunction directly contributes to atrial remodeling—encompassing both structural and electrical alterations—by disrupting myocardial energy homeostasis [13]. Concurrently, it exacerbates inflammatory processes and oxidative stress, thereby promoting the progression of atrial fibrillation [14]. Diabetes mellitus, a prevalent clinical manifestation of metabolic disease, constitutes a well-established independent risk factor for AF [15]. Furthermore, diabetes is associated with worsened clinical outcomes in AF patients, including a greater symptom burden, impaired quality of life, increased hospitalization rates, and elevated mortality. Insulin resistance (IR), a pathological state where tissue has decreased sensitivity to insulin, is the initiating link in type 2 diabetes. Existing studies have demonstrated that IR is also an important risk factor for cardiovascular disease, including AF [16,17]. Therefore, assessing the IR levels can help to predict the risk of developing AF and disease progression. The previous gold standard for assessing IR, the glucose clamp technique [18], has limited clinical applications due to its complexity, time-consuming nature, and high cost. Recent studies have shown that the triglyceride–glucose index (TyG index) is an easy-to-use, low-cost and clinically valuable alternative assessment tool for IR [19,20,21]. The TyG index is calculated using the fasting triglyceride and blood glucose levels: TyG index = Ln [TG (mg/dL) × FBG (mg/dL) ÷ 2] [19]. Several studies have linked an elevated TyG index to an increased risk of several cardiovascular events, including atherosclerosis and myocardial infarction [22,23]. A meta-analysis suggested that the TyG index was higher in an AF population than a no-AF population and has potential for use as an AF risk prediction tool [24].

This narrative review synthesizes recent evidence on the association between the TyG index and AF, outlines plausible indirect pathophysiological pathways, and discusses potential clinical applications, the current challenges, and future research priorities in AF risk assessment.

## 2. Pathophysiologic Pathways Linking Insulin Resistance to Atrial Fibrillation

Although the precise mechanisms of AF remain incompletely understood, a growing body of research emphasizes the central role of IR in atrial remodeling [16,25]. IR helps to promote the increased vulnerability of the electrophysiological substrate and anatomical remodeling foundational to AF development by disrupting myocardial energy metabolism, interfering with ion channel function, activating inflammatory pathways, and damaging cellular structures. These processes align closely with the current international consensus on the mechanisms of ACM.

At the molecular level, IR impairs GLUT4 and GLUT8 transport within cardiomyocytes [25], leading to a reduced glucose uptake and gradual shift in the cardiac energy production process from glucose oxidation to fatty acid metabolism. Such metabolic reprogramming induces the accumulation of lipotoxic products (e.g., diacylglycerol, ceramides), leading to reduced ATP production efficiency. This is accompanied by increased mitochondrial and NADPH oxidase activity, resulting in excessive reactive oxygen species (ROS) production [26,27]. ROS and chronic low-grade inflammation cause oxidative stress, promoting phosphorylation of CaMKIIδ (calcium/Calmodulin-dependent Protein Kinase II delta). This leads to excessive phosphorylation of RyR2 (Ryanodine Receptor 2) and PLB (Phospholamban), thereby enhancing calcium leakage from the sarcoplasmic reticulum (SR) during diastole. This triggers delayed afterdepolarizations (DADs) and ectopic excitation [16]. Simultaneously, inflammatory mediators and ROS downregulate the expression of INa (fast sodium current), Ica (L-type calcium current), and the gap junction proteins Cx40 and Cx43 [28], slowing atrial conduction and increasing the electrical heterogeneity. This provides the electrophysiological basis for AF initiation and reentry circuits. Chronic inflammation induced by IR and hyperinsulinemia can activate the sympathetic–renin–angiotensin–aldosterone system (RAAS) axis, causing an autonomic imbalance, shortening the effective refractory period (ERP), and increasing the sensitivity of atrial muscle to triggering events.

At the cellular level, impaired metabolic substrate conversion and mitochondrial dysfunction within cardiomyocytes lead to there being insufficient intracellular energy and disrupted calcium homeostasis, resulting in increased triggering activity and susceptibility of the refractory period to changes. IR also promotes excessive accumulation of epicardial fat, where adipocytes synergize with ROS to enhance secretion of pro-inflammatory/pro-fibrotic factors such as TNF-α (Tumor Necrosis Factor-alpha), IL-6 (Interleukin-6), TGF-β1 (Transforming Growth Factor-beta 1), and MMP-9 (Matrix Metalloproteinase-9). This further activates fibroblasts, increases collagen synthesis, and leads to interstitial fibrosis [29]. Endothelial dysfunction and microcirculatory injury amplify inflammatory/fibrotic signaling, further enhancing electro-matrix remodeling [19].

Collagen deposition and interstitial fibrosis increase the atrial wall’s stiffness, forming conduction block zones and low-voltage areas that provide the anatomical and electrophysiological substrate for reentrant circuits. Left ventricular diastolic dysfunction associated with IR, coupled with a left atrial pressure–volume overload, leads to atrial enlargement and increased atrial wall tension, thereby promoting structural remodeling. Slower conduction, enhanced electrophysiological heterogeneity, and shortened action potential refractory periods synergistically lower the reentry threshold and stabilize AF circuits. Overall, IR continuously shapes the underlying electro-structural vulnerability and ACM phenotype in AF through “metabolic reprogramming and calcium imbalance–inflammation/fibrosis-driven matrix alterations–conduction slowing and shortened refractory period,” at levels extending from the molecular and cellular to the clinical–pathological [16,19,28,29]. Accordingly, identifying IR states and intervening holds promise as a critical strategy for preventing the onset of AF and halting ACM progression.

**Figure 1:**
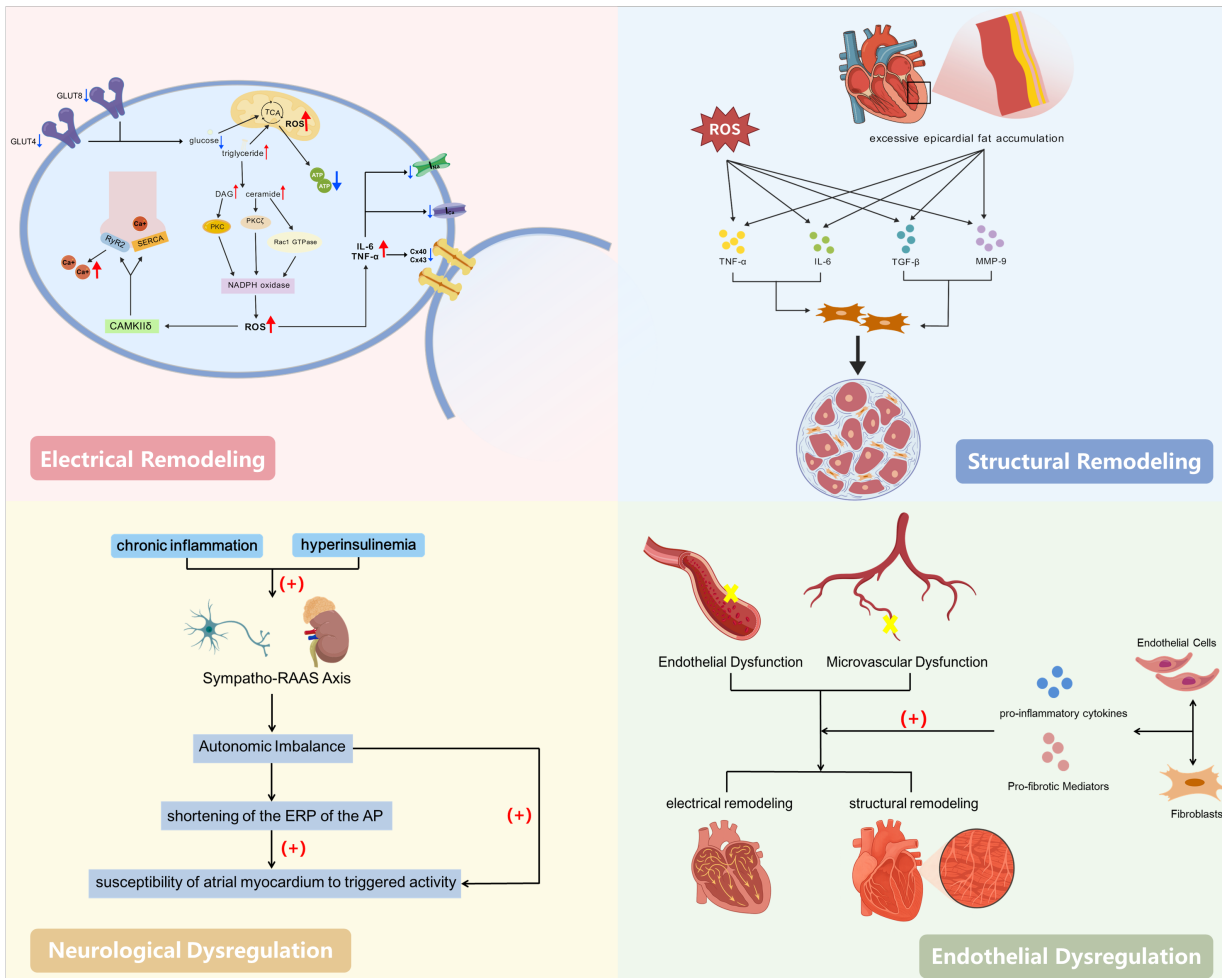
Mechanisms underlying the association between insulin resistance and atrial fibrillation. Created with BioGDP.com [33].

In terms of metabolic phenotypes, dyslipidemia and impaired glucose metabolism represent the two core hallmarks of IR: reduced insulin-mediated suppression of lipolysis leads to elevated circulating triglyceride (TG) levels, while diminished insulin-mediated glucose uptake in the muscles and liver results in increased fasting blood glucose (FBG) levels. The TyG index, as the logarithmic transformation of their product, sensitively reflects the degree of IR. Extensive research indicates that compared to the HOMA-IR index derived from steady-state models, the TyG index serves as an alternative indicator for assessing IR [20,21,30,31] and correlates with multiple cardiovascular diseases [32]. Therefore, as a simple and cost-effective alternative indicator, further exploration of the relationship between the TyG index and AF in clinical practice holds significant importance. A specific description of the mechanisms is provided in Figure 1.

## 3. Association of Triglyceride–Glucose INDEX with Risk of Atrial Fibrillation

### 3.1. Triglyceride–Glucose INDEX and the Incidence of Atrial Fibrillation

Based on evidence from several studies, the TyG index, a core marker of IR, has a significant and complex association with the AF risk in different populations, and its predictive value is moderated by an individual’s metabolic status, gender, and other factors. Most studies confirm that an elevated TyG index is an independent risk factor for AF. In a population with type 2 diabetes, the risk of AF increased by 40.6% for every increase in one standard deviation (SD) in the TyG index [odds ratio (OR) = 1.406], exhibiting a linear, dose-dependent relationship [34]. The association was stronger in a population without diabetes (OR = 3.065) [35], while significant positive associations were shown in hypertension (OR = 1.957) [36], acute coronary syndrome (ACS) (OR = 2.02) [37], and patients with non-alcoholic fatty liver disease (NAFLD) (OR = 4.84) [38]. However, a U-shaped association between the TyG index and AF was found in the general population without cardiovascular disease and a large UK Biobank sample population, with a minimum risk interval of 8.80–9.20 [39,40]. Both low and high TyG indexes increased the risk [hazard ratio (HR) = 1.15 in the low-TyG-index group and HR = 1.18 in the high-TyG-index group], and this U-shaped curve persisted in women and was not significant in men [39]. The strong association between a high TyG index and an increased risk of AF may be due to the IR status, whereas an increased risk of AF due to a low TyG index may be caused by low fasting glucose. Hypoglycemia may be a manifestation of abnormalities in the nutritional and metabolic status [41,42], and a large number of previous studies have found that low blood glucose levels are associated with a higher risk of all-cause mortality, stroke, cardiovascular events, etc. [43,44,45,46]. From the above studies, a certain correlation can be seen between the TyG index and AF, but different studies vary in their conclusions. Therefore, future studies need to further explore the mechanisms underlying the relationship between the TyG index and AF and its moderation by a combination of different factors. A description of the specific studies examined is provided in Table 1.

### 3.2. Triglyceride–Glucose Index and Recurrence of Atrial Fibrillation After Ablation

Cardiac radiofrequency ablation (RFA) is a key treatment for AF and uses radiofrequency energy to restore the heart to a regular rhythm [47,48]. Recurrence of AF occurs in 30% to 40% of patients after the first procedure [49], with the risk factors being a significant increase in the left atrial volume, advanced age, AF for a prolonged duration, renal insufficiency, and other cardiovascular risk factors [50]. Determining how to accurately and objectively predict the recurrence of AF before surgery and provide optimal treatment is very important, but there is still no strong predictive index for AF recurrence.

To explore the use of a new index to stratify the risk of AF recurrence, some scholars conducted a study on the predictive value of the TyG index regarding the risk of late AF recurrence after RFA in patients without diabetes, and the results demonstrated that it was an independent risk factor and had good predictive value [Area Under the Curve (AUC) = 0.737] [51]. Others compared the ability of four non-insulin-dependent IR indices to predict AF recurrence after RFA [the Metabolic Score for Insulin Resistance (METS-IR), TyG, TyG-BMI, and Triglyceride-to-HDL-Cholesterol Ratio (TG/HDL-C Ratio)] and found that the TyG, METS-IR, and TyG-BMI indices were independently associated with the risk of AF recurrence after surgery (HRs were 1.18, 1.82, and 1.71 after adjusting for confounders; all *p*-values were <0.05), with the TyG-BMI index having the highest predictive power (AUC = 0.608) [52]. The clinical application of the TyG index in assessing the recurrence risk after RFA was further extended by a recent study based on a machine learning approach, in which a prediction model was constructed using the preoperative TyG index and the amount of left atrial epicardial fat and peripheral fat around the left echogenic branch determined using coronary CT angiography, with the TyG index being the most important feature variable in the model [53]. This further suggests that metabolic–structural coupling mechanisms have an important role in AF recurrence and that combining consideration of the TyG index and traditional AF risk factors may provide greater value in clinical practice.

As a metabolic assessment parameter based on the FBG and TG levels, the components of the TyG index show dynamic fluctuations in response to changes in the individual’s physiological state and the passage of time. The analysis in traditional studies using the TyG index values at a single point in time has two significant limitations: first, it is difficult to completely reflect the temporal evolution of this indicator, which undermines its value for use in dynamic monitoring; second, it is not possible to effectively assess the long-term efficacy of clinical interventions (e.g., lipid or glycemic management programs) developed in response to TyG index abnormalities. To break through this methodological bottleneck, recent research paradigms have shifted to using novel assessment systems such as the TyG index trajectory and cumulative TyG exposure. These integrate longitudinal data collected at multiple time points to investigate the mechanism underlying the association between TyG indices and the progression of metabolic diseases from a dynamic monitoring perspective [54,55]. Jia et al. conducted a large-scale, multicenter retrospective study that included 997 patients with stage 3D AF, and for the first time, they investigated the predictive value of changes in the TyG index trajectory during the postoperative “blanking period” regarding the recurrence of AF. The study used a latent class trajectory model to categorize the patients into three groups—a persistently low-level TyG group, a fluctuating group, and a persistently high-level group—and the results showed that the patients in the persistently high-level group had a significantly higher risk of recurrence than those in the persistently low-level group [54]. In addition, Yan et al. designed a retrospective cohort study of 576 patients with AF who underwent RFA for the first time. Their fasting triglyceride and glucose levels were measured preoperatively and at 1 month and 3 months postoperatively to calculate their TyG indices, which were time-weighted and aggregated to obtain the “cumulative TyG index”. The patients were then categorized into three groups: low, medium, and high TyG. The findings suggested that the AF recurrence rate increased significantly with the cumulative TyG index, with a recurrence rate of 4.0% in the low-level group and up to 32.8% in the high-level group. Multivariate Cox regression further verified that the risk of recurrence was significantly higher in the high-level group than the low-level one (HR = 8.716) [55]. These findings suggest that early postoperative control of patients’ glucose and lipid levels may be a key strategy to improve their outcomes after ablation. A description of the specific studies examined is provided in Table 2.

### 3.3. Triglyceride–Glucose Index and New-Onset Atrial Fibrillation After Cardiac Surgery

New-onset AF (NOAF) after cardiac surgery is defined as new-onset AF with a duration of >30 s after cardiac surgery in a patient with no previous history of AF, usually occurring on the second day after cardiac surgery [56]. NOAF after cardiac surgery is a common complication, associated with increased risks of mortality and stroke, with an overall incidence of 30 to 60 percent [57]. Predictive markers identified in previous studies include homologous chimeras, acetylglutamine, ornithine, methionine, and arginine [58,59]. Recent studies have focused on the predictive value of the TyG index in patients with NOAF after cardiac surgery. Wei et al. performed a retrospective analysis of 409 patients with hypertrophic obstructive cardiomyopathy (HOCM) who underwent septal myectomy and showed that the TyG index was an independent predictor of NOAF, with an AUC of 0.723 obtained in the Receiver Operating Characteristic (ROC) curve analysis. Further comparison of the traditional prediction models’ discriminative abilities before and after incorporating the TyG index revealed that the AUC improved from 0.742 to 0.793 (*p* = 0.065), an increase that was not significant but suggests that the TyG index has potential complementary value in clinical risk stratification [60] The TyG index has been demonstrated to be an independent predictor of NOAF after a percutaneous coronary intervention (PCI) in patients with ST-segment elevation myocardial infarction (STEMI). Multifactorial regression analysis showed that the risk of NOAF increased 8.884-fold for every one-unit increase in the TyG index, with significant predictive efficacy (AUC = 0.758) and an optimal cutoff value of 9.15 (sensitivity of 71.43%, specificity of 73.77%) [61]. Similar results were obtained in another study, where a prediction model based on the TyG index was effective in identifying the group with a high risk of NOAF after PCI treatment in patients with acute myocardial infarction (AMI) [62]. In a cohort study of 542 patients undergoing off-pump coronary artery bypass grafting (OPCABG), Peng et al. demonstrated the use of the CT-measured visceral adiposity index (VAI) and TyG index as novel metabolic predictive markers, which independently predicted NOAF after non-extracorporeal coronary artery bypass grafting (CABG). The study showed that patients with a high VAI (>51.34 cm^2^/m^2^) and TyG index had a significantly increased risk of NOAF (highest tertile HRs of 2.58 and 2.88, respectively), and both markers had significant synergistic predictive value when combined with a traditional risk model (joint AUC of 0.897), providing a tool for the quantitative metabolism- and fat-distribution-based preoperative identification of patients at a high risk of NOAF [63]. A description of the specific studies examined is provided in Table 3.

### 3.4. Triglyceride–Glucose Index and Prognosis in Atrial Fibrillation Patients

AF significantly increases the risk of adverse outcomes such as death, heart failure, hospitalization, and thromboembolic events [64,65]. Based on a prospective cohort analysis (n = 1979), Yin et al. demonstrated for the first time that the TyG index is an independent predictor of major cardiovascular and cerebrovascular events (MACCEs) in patients with AF. The group in the highest TyG index quartile (>9.06) had a significantly increased risk of MACCEs (HR = 2.103), which was more pronounced in younger patients (<60 years) (HR = 3.927), suggesting that the TyG index could be used as a novel metabolic marker for risk stratification of AF patients [66]. One study also found an S-shaped dose–response relationship between the TyG index and major adverse cardiovascular events (MACEs) by analyzing a retrospective cohort of 864 AF patients without diabetes (critical window of 8.715–9.725). The risk of MACEs was significantly increased in the high-TyG-index group (≥9.023) [67]. These two different conclusions may have been influenced by the characteristics of the target population, interference from the interventions, and differences in research methods. However, it is noteworthy that despite their different conclusions, the two studies revealed the same pattern, i.e., that the prognosis of patients with AF significantly worsened when the TyG index exceeded a certain threshold. Studies have also shown a significant positive correlation between the TyG index and short-term mortality in patients with critical AF [68,69]: in 1146 intensive care unit (ICU) patients with AF, Ma et al. found that the 30-day risk of death in the highest TyG index quartile (>9.24) increased by 71% compared to that in the group with the lowest index. Furthermore, Kan et al. revealed that when AF occurred alongside heart failure, the increase in this risk rose to 167%, and the risk of ICU mortality surged by 289%. Together, these two studies confirm that the TyG index can be used as an early warning indicator for the risk of death in ICU patients with AF. A description of the specific studies examined is provided in Table 4.

### 3.5. Heterogeneity and Effect Modification

Although IR is an important pathologic basis for diabetes, IR alone is not sufficient to confirm a diagnosis of diabetes. Progression to diabetes also depends on the ability of pancreatic beta cells to compensate for insulin secretion. The pattern of the association between the TyG index and risk of AF also varies among populations with different glycemic statuses. In patients with diabetes mellitus, a linear positive correlation between the TyG index and AF risk has been reported [34], while others have found an inverse L-shaped relationship, with a significant increase in the risk only when the TyG index reaches a high level [40], and some studies have not observed a significant association between the two [35]. In a population without diabetes, the same positive correlation [35] and U-shaped association [40] were found. A mechanism underlying the increased risk of AF in low-TyG-index groups may be hypoglycemia. Firstly, hypoglycemia over-activates the sympathetic–adrenal axis, leading to a prolonged QT interval and myocardial electrophysiological instability. Secondly, hypoglycemia causes hypokalemia or hypomagnesemia through inhibition of renal sodium reabsorption, interferes with atrial repolarization, and increases the susceptibility to cardiac arrhythmias. Thirdly, when the energy supply is insufficient, the function of cardiomyocyte sodium–potassium pumps is impaired, leading to the accumulation of intracellular sodium ions and disruption of calcium transients, which ultimately enhances triggering activity [40]. In an HOMA-IR-based study, Lee et al. found that when the HOMA-IR was in the range of 1–2.5, the risk of AF increased with it; however, when the HOMA-IR was <1 or >2.5, the change in the risk of AF was not significant [30]. This phenomenon suggests that the positive correlation between IR and AF stabilizes after reaching a certain threshold, which may be related to the irreversible structural remodeling of the atria in patients with diabetes, thus making the “protective” effect of a low TyG index less likely to be seen in this population. In addition, the association between the TyG index and AF may also vary by gender, and this may arise from differences in factors such as fat distribution, baseline smoking and drinking habits, and substance use [39].

Additionally, studies have found that South Asian populations exhibit higher IR and metabolic syndrome risk, along with elevated TG levels, yet their AF prevalence remains lower than that of European Caucasians [70]. This discrepancy may be attributed to the following factors. First, South Asian populations may exhibit certain genetic differences compared to other populations, which could potentially influence susceptibility to IR, metabolic syndrome, and AF. Second, South Asian populations exhibit a higher risk of metabolic syndrome, but in certain regions, traditional dietary patterns and lifestyles may confer protective effects against AF [3].

### 3.6. Other Non-Insulin-Based Insulin Resistance Indices

Simple and low-cost biomarkers hold significant clinical importance, enabling improved risk stratification, prognosis assessment, and the ability to guide treatment strategies. In addition to the TyG index, researchers have explored other non-insulin-based indices of IR. Specific metrics and their calculation formulas are shown in Table 5. A recent systematic review and meta-analysis investigated whether these indicators can predict the risk of AF recurrence following ablation. Based on the evidence from this study, various non-insulin-based IR indices, particularly the TyG index and METS-IR, possess independent predictive value for post-ablation recurrence. Their effect direction and magnitude remained consistent across multiple studies. However, the lack of uniform thresholds and the limited geographical scope of the research restrict its generalizability. Future validation of these indices’ incremental value and effects on reclassification through multicenter prospective studies is required [71].

## 4. Potential Clinical Implications

**Figure 2:**
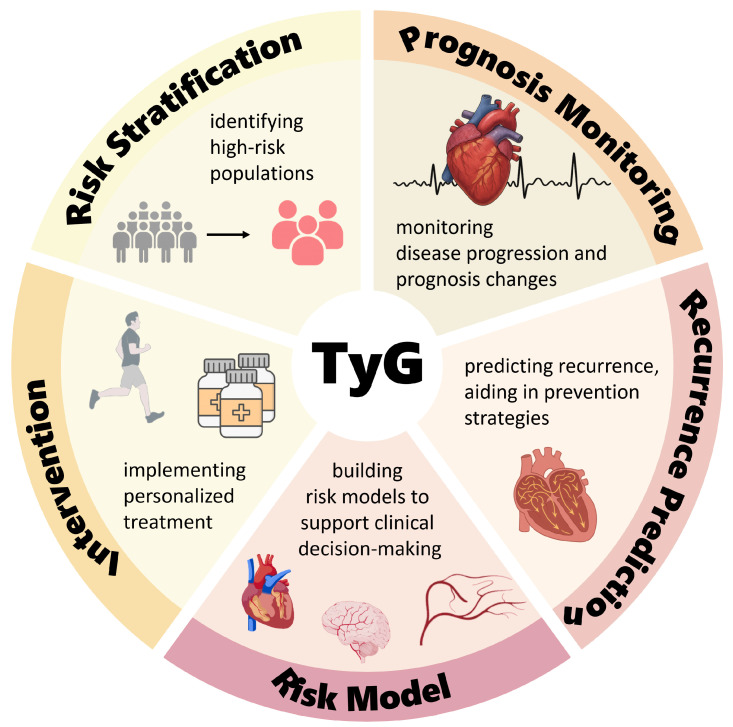
Clinical application prospects of the triglyceride–glucose index. Created with BioGDP.com [33].

IR is a common pathological basis of many metabolic diseases, and its harmful effects go beyond increasing the risk of diabetes, including damage caused by “metabolic toxins” quietly attacking multiple organ systems throughout the whole body. There have also been a number of studies demonstrating that IR increases the risk of cardiovascular disease [72]. IR-induced metabolic remodeling directly affects the development of the AF substrate and ACM through multiple mechanisms and signaling pathways. Although a hyperinsulinemic–euglycemic clamp is the gold standard for assessing the IR status, it is difficult to generalize its use in routine clinical practice due to this procedure’s extreme complexity, time-consuming nature—taking up to several hours—high cost, and risk of hypoglycemia. As an alternative to this gold standard, the HOMA-IR is used as a core tool for epidemiologic screening and providing primary care due to its simplicity and low cost. However, it is significantly dependent on the use of standardized insulin assays and grossly underestimates the degree of IR in advanced diabetic β-cell failure with reduced fasting insulin, as well as being inappropriate for patients treated with insulin. In addition, it does not reflect the dynamic metabolic state after meals [73]. Compared with the above two methods, measurement of the TyG index requires only basic lipid and glucose testing, completely avoids the standardization of insulin measurement and interference from the β-cell function, and is simple, low-risk, and highly stable, providing a cost-effective screening solution for use in primary care to identify potential AF patients as early as possible. Based on the risk factors for and pathological mechanisms of ACM, the TyG index can also be combined with the BMI, inflammatory markers, left atrial volume, epicardial fat, P-wave dispersion, etc., to form a “metabolic–structural–electrophysiological” triple prediction model. This can significantly optimize the risk stratification of patients with atrial fibrillation, allow for a focus on the diagnosis and treatment of high-risk patients, and provide individualized treatment plans for patients. The TyG index can also be integrated into existing risk scoring criteria, such as $CHA_{2}DS_{2}-VASc$, to optimize the screening efficiency in high-risk populations and provide a time window for individualized metabolic interventions to ultimately reduce atrial remodeling-related cardiovascular events. Changes in the components of the TyG index, the fasting triglyceride and blood glucose levels are reversible, and the onset or recurrence of AF can be prevented by instructing patients in exercise, dietary management, and medication. Clinical potential applications of the TyG index are illustrated in Figure 2.

Additionally, the accessibility of the TyG index measurement makes it highly suitable for use as a dynamic monitoring indicator to evaluate the effectiveness of interventions. However, no unified guidelines currently exist. The following approach can be used to fully leverage the TyG index for assessment purposes. Under standardized conditions (8–12 h of fasting, uniform laboratory conditions and time points), the fasting TG and FBG levels should be regularly measured to calculate the TyG index. Baseline stratification should be performed using population (or center-specific) percentiles, and the follow-up frequencies should be set according to the risk levels (e.g., high-risk patients should be followed up every 3 months, moderate-risk patients every 6 months, and low-risk patients every 12 months; additional measurements should be taken perioperatively and at 1–3 months post-ablation). At each follow-up visit, in addition to recording the current TyG levels, it is recommended to concurrently assess the relative change (ΔTyG%), long-term trend (annual slope), and variability (SD/CV or VIM) in these levels and the time-weighted/cumulative TyG index to reflect the exposure burden and metabolic stability. When the TyG index reaches or remains at high levels, or when the ΔTyG% shows a significant increase or a persistently upward trajectory, management actions should be triggered. These include intensified ECG monitoring and enhanced weight/exercise/nutrition interventions for insulin resistance. Perioperative rhythm and electrolyte management may be intensified, with increased follow-ups and re-evaluations for re-intervention after ablation. Conversely, if the ΔTyG% decreases and remains consistently low or shifts to a lower percentile, the follow-up frequency may be appropriately reduced. The intervention’s effects can be assessed by evaluating the early ΔTyG% at 8–12 weeks post-initiation, reviewing its trajectory at 6 months, and interpreting the results in conjunction with the patient’s body weight, HbA1c, inflammatory markers, left atrial indices, and AF burden. When necessary, the TyG index should be incorporated into a comprehensive model alongside $CHA_{2}DS_{2}-VASc$ scores and imaging/inflammatory markers for refined stratification. It should be noted that the current evidence is primarily observational. The TyG index should be used for risk stratification and management decision support rather than as a diagnostic threshold. Its threshold values are population-specific and should be interpreted in the clinical context and in accordance with the current specialist consensus.

## 5. Limitations and Directions

The existing clinical evidence linking the TyG index to AF primarily consists of retrospective cohort studies with diverse samples and settings. The exposure is often assessed using the TyG index at a single time point, though the recently adopted TyG index trajectory/cumulative TyG index better captures the sustained metabolic burden. The outcome definitions (incidence, recurrence, postoperative NOAF, and mortality/MACCEs) are relatively standardized, but the follow-up periods vary. Although confounder adjustment is commonly performed, inconsistent handling of metabolic-related medications and fat distribution limits the discriminatory power of the TyG index as a single marker, making it more suitable for inclusion in multivariate models. Heterogeneity primarily stems from population differences (patients with T2DM, hypertension, ACS, NAFLD, etc., versus the general population; a U-shaped relationship is also observed in the general population, with a greater prominence in women), exposure characterization methods (quantile/threshold, single time point vs. trajectory/cumulative), and differences in the outcome scenarios (incidence, recurrence after ablation, perioperative NOAF, long-term prognosis). These factors contribute to inconsistencies in the effect sizes and magnitude of the benefits.

Given the aforementioned limitations, future research in this area can be improved with regard to three aspects: the methodology, mechanisms investigated, and clinical translation. Methodologically, future research should employ multicenter, prospective, and pre-registered studies; standardize laboratory testing and event definitions; employ repeated measurements to characterize the TyG index trajectories and cumulative exposure; identify optimal cutoff values and dose–response relationships for both universal and context-specific scenarios; and perform stratified validation by age, sex, and metabolic phenotype. In terms of modeling and mechanisms, separate pathways have been established for NOAF, post-ablation recurrence, perioperative non-operative AF, and the long-term prognosis to construct models, standardize reporting of incremental values and calibration, and identify actionable targets. This has been achieved by integrating quantification of the metabolic burden and inflammation, cardiac structural remodeling, and electrical remodeling with multimodal imaging and longitudinal mediation/causal analysis. Regarding intervention and translation, SGLT2 inhibitors have been demonstrated to significantly reduce the TyG index [72], while GLP-1 receptor agonists (GLP-1RAs) show potential for use in treating cardiometabolic complications [74]. However, there remains insufficient evidence from research to confirm whether antidiabetic and lipid-lowering drugs can reduce the risk of AF or their therapeutic efficacy. Randomized or quasi-experimental studies can be conducted using the TyG index as a modifiable risk marker to evaluate the reversibility of glucose-lowering/lipid-modifying strategies (e.g., employing SGLT2 inhibitors, GLP-1 receptor agonists, metformin, and statins) and their impact on AF’s occurrence and recurrence and NOAF. Additionally, the TyG index can be integrated with structural imaging, adiposity metrics, and traditional risk factors to develop interpretable risk scores and online tools. These should undergo external validation and multicenter calibration before being embedded into perioperative/ablation workflows. In terms of data and implementation, we could fully integrate laboratory, imaging, and electrophysiological data to construct a time-series database. By combining wearable technology with AI, we could develop a dynamic early warning system and achieve subtype classification and personalized metabolic intervention. Real-world studies could evaluate this system’s cost-effectiveness and resource allocation, validating the value of a closed-loop “screening–intervention–reassessment” system in improving outcomes. This would provide evidence for guideline updates and “precision metabolic regulation.”

In addition to traditional IR surrogate markers, recent studies have suggested that emerging metabolic factors may play a role in the development and progression of AF. For example, irisin, a myokine secreted by skeletal muscle, is closely associated with energy metabolism regulation, insulin sensitivity, and cardiovascular remodeling [75,76,77,78]. Some studies suggest that alterations in irisin levels may be associated with AF risk, but existing evidence remains limited and the mechanisms are not fully elucidated [79,80]. Therefore, it is necessary to further investigate the role of metabolic factors such as irisin in large-scale population studies and basic experiments. This should be combined with comprehensive assessments using IR indicators like TyG to establish a more robust metabolic-cardiac remodeling-AF risk prediction system.

## 6. Conclusions

The available evidence tentatively supports the value of the TyG index in predicting the risk of AF, but the evidence for a causal association is still limited, and validation through large-sample prospective cohort studies is urgently needed. If an association is established, clinical application of the TyG index is expected to reduce the AF morbidity and associated mortality, improve patients’ quality of life, and reduce the economic burden on the healthcare system.

## Figures and Tables

**Table 1:** Studies on TyG index and incidence of atrial fibrillation.

Study	Population	Design	Total Participants	Participants with AF	TyG Index Groups	Cutoff Value	HR/OR (95% CI)	References
Li et al., 2025	Hypertensive patients	Retrospective cohort	566	306	Not reported	Not reported	Continuous variable OR of 1.957 (1.452–2.639)	[36]
Shanshan Shi et al., 2025	Individuals aged 37–73 years with no history of heart disease	Retrospective cohort	409,705	26,092	Tertiles	Not reported	T1 vs. T2 HR of 1.22 (1.17–1.27) T3 vs. T2 HR of 1.09 (1.05–1.14)	[40]
Yao et al., 2024	Patients with ACS	Retrospective cohort	613	70	Q1 (≤1.10) Q2 (1.10–1.64) Q3 (1.64–2.25) Q4 (>2.25)	Not reported	Continuous variable OR of 2.02 ( 1.51–2.71) Q4 vs. Q1 OR of 3.16 (1.02–9.83)	[37]
Liu et al., 2023	Individuals aged 45–64 years with no history of heart disease	Prospective cohort study	11,851	1925	T1 (<8.80) T2 (8.80–9.20) T3 (>9.20)	Not reported	T1 vs. T2 HR of 1.15 (1.02–1.29) T3 vs. T2 HR of 1.18 (1.03–1.37)	[39]
Zhang et al., 2023	Patients with NAFLD	Retrospective cohort	912	204	Q1 ≤ 8.39 Q2 ≤ 8.78 ± 0.83 ± 0.20 Q3 ≤ 9.09 ± 0.99 Q4 ≤ 9.57 ± 0.27	Not reported	Continuous variable OR of 4.84 (2.98–7.88) T4 vs. T1 OR of 4.34 (2.37–7.94)	[38]
Chen et al., 2022	Hospitalized patients	Retrospective observational study	358	179	Not reported	8.35	Continuous Variable OR 2.092 (1.412–3.100)	[35]
Wenrui Shi et al., 2022	Patients with T2DM	Cross-Sectional Observational Study	3244	213	Quartiles	Not reported	Continuous variable OR of 1.406 (1.197–1.650) Q4 vs. Q1 OR of 2.120 (1.303–3.348)	[34]

ACS: acute coronary syndrome; NAFLD: non-alcoholic fatty liver disease; T2DM: type 2 diabetes mellitus; AF: atrial fibrillation; TyG index: triglyceride–glucose index; HR: hazard ratio; OR: odds ratio; CI: confidence interval.

**Table 2:** Studies on TyG index and recurrence of atrial fibrillation after ablation.

Study	Population	Design	Total Participants	Recurrence	TyG Index Groups	Cutoff Value	HR (95% CI)	References
Li et al., 2024	Patients undergoing RFCA	Retrospective cohort	325	79	Not reported	Not reported	Continuous variable HR of 2.268 (1.372–3.750)	[53]
Jia et al., 2024	Patients with stage 3D AF	Retrospective cohort	997	200	T1 ≤ 8.67 T2 8.67–9.37 T3 > 9.37	Not reported	Continuous variable HR of 1.255 (1.087–1.448) T3 vs. T1 HR of 2.056 (1.335–3.166)	[54]
Yan et al., 2024	Patients undergoing RFCA	Retrospective cohort	375	67	T1 < 2.07 T2 2.07–2.14 T3 ≥ 2.14	2.11	T2 vs. T1 HR of 4.949 (1.778–13.778) T3 vs. T1 HR of 8.716 (3.371–22.536)	[55]
Wang et al., 2024	Patients undergoing RFCA	Retrospective cohort	2242	711	T1 ≤ 8.325 T2 8.325–8.765 T3 > 8.765	8.692	T3 vs. T1 HR of 1.25 (1.03–1.51)	[52]
Tang et al., 2022	Patients without diabetes undergoing RFCA	Retrospective cohort	275	70	T1 < 6.08–8.67 T2 8.68–9.37 T3 ≥ 9.38	9.24	Continuous variable HR of 2.015 (1.408–4.117)	[51]

RFCA: radiofrequency catheter ablation; AF: atrial fibrillation; TyG index: triglyceride–glucose index; CI: confidence interval.

**Table 3:** Studies on TyG index and new-onset atrial fibrillation after cardiac surgery.

Study	Population	Design	Total Participants	NOAF Incidence	TyG Index Groups	Cutoff Value	HR/OR (95% CI)	References
Wu et al., 2025	Patients with AMI (PCI)	Retrospective cohort	551	94	Not reported	Not reported	Continuous variable OR of 1.981 (1.344–2.92)	[62]
Peng et al., 2023	Patients with AMI (OPCABG)	Retrospective cohort	542	Not reported	Q1 ≤ 8.45 Q2 8.45–8.80 Q3 > 8.80	8.99	Continuous variable HR of 1.24 (1.03–1.73) Q3 vs. Q1 HR of 2.88 (1.76–4.71)	[63]
Ling et al., 2022	Patients with AMI (PCI)	Retrospective cohort	549	42	Not reported	9.15	Continuous variable OR of 8.884 (1.570–50.265)	[61]
Wei et al., 2021	Patients with HOCM (septal myectomy)	Retrospective cohort	409	61	Not reported	7.60	Continuous variable OR of 4.218 (2.381–7.473)	[60]

AMI: acute myocardial infarction; PCI: percutaneous coronary intervention; OPCABG: off-pump coronary artery bypass grafting; HOCM: hypertrophic obstructive cardiomyopathy; TyG index: triglyceride–glucose index; HR: hazard ratio; OR: odds ratio; CI: confidence interval.

**Table 4:** Studies on TyG index and prognosis in atrial fibrillation patients.

Study	Population	Design	Total Participants	Prognosis	TyG Index Groups	HR/OR (95% CI)	References
Kan et al., 2025	Patients with AF and CHF	Retrospective cohort	787	Hospital mortality: 112 ICU mortality: 65	Q1 7.21–8.46 Q2 8.46–8.84 Q3 8.84–9.30 Q4 9.30–13.49	Hospital mortality: Continuous variable OR of 1.59 (1.15–2.19) Q4 vs. Q1 OR of 2.67 (1.30–5.50) ICU mortality: Continuous variable OR of 1.90 (1.28–2.83) Q4 vs. Q1 OR of 3.89 (1.50–10.07)	[69]
Ma et al., 2025	Patients with critical AF	Retrospective cohort	1146	All-cause mortality: 7-day: 142 15-day: 214 30-day: 278	Q1 < 8.41 Q2 8.41–8.77 Q3 8.77–9.24 Q4 > 9.24	7-day mortality: Continuous variable HR of 1.48 (1.15–1.90) Q4 vs. Q1 HR of 2.40 (1.38–4.18) 15-day mortality: Continuous variable HR of 1.35 (1.09–1.67) Q4 vs. Q1 HR of 2.02 (1.30–3.31) 30-day mortality: Continuous variable HR of 1.36 (1.13–1.65) Q4 vs. Q1 HR of 1.71 (1.17–2.49)	[68]
Gong et al., 2025	Patients with AF without diabetes	Retrospective cohort	864	MACEs: 148	T1 ≤ 8.493 T2 8.494–9.022 T3 ≥ 9.023	Continuous variable HR of 1.77 (1.44–2.17) T3 vs. T1 HR of 1.91 (1.53–2.38)	[67]
Yin et al., 2024	Patients with AF	Retrospective cohort	1979	MACCEs: 227	Q1 6.80–8.21 Q2 8.21–8.59 Q3 8.59–9.06 Q4 9.06–11.81	Q4 vs. Q1 HR of 2.103 (1.107–3.994)	[66]

AF: atrial fibrillation; CHF: congestive heart failure; ICU: intensive care unit; MACEs: major adverse cardiovascular events; MACCEs: major cardiovascular and cerebrovascular events; TyG index: triglyceride–glucose index; HR: hazard ratio; OR: odds ratio; CI: confidence interval.

**Table 5:** Other non-insulin-based insulin resistance indices.

Index	Calculation Formula	Combined Effect	Heterogeneity	Direction and Magnitude	Interpretation of Evidence
TyG	Ln[TG(mg/dL) × FBG(mg/dL)/2]	HR of 1.29 (1.15–1.44)	I${⁠}^{2}$ = 44%	The higher the index, the greater the risk of recurrence	The hazard ratio of the TyG index is the highest among the non-insulin-based insulin resistance indices, and calculation of this index can be prioritized for baseline stratification
METS-IR	Ln[(2 × FBG) + TG] × BMI ÷ Ln[HDL-C]	HR of 1.04 (1.03–1.05)	I${⁠}^{2}$ = 0%	Small increase in risk per unit increase	Small variation but strong consistency
TG/HDL	TG ÷ HDL-C	HR of 1.09 (0.96–1.24)	I${⁠}^{2}$ = 86%	The direction is consistent but not significant	Insufficient evidence for use as a predictor alone
TyG-BMI	TyG × BMI	Insufficient data pooled	-	-	More prospective studies are needed
eGDR	19.02 − (0.22 × BMI) − (3.26 × hypertension) − (0.61 × HbA1c)	Insufficient data pooled	-	-	Single research report only

## Abbreviations

The following abbreviations are used in this manuscript:

AF	Atrial fibrillation
ACM	Atrial cardiomyopathy
IR	Insulin resistance
TyG index	Triglyceride–glucose index
GBD	Global Burden of Disease
ROS	Reactive oxygen species
CaMKIIδ	Calcium/Calmodulin-dependent Protein Kinase II delta
RyR2	Ryanodine Receptor 2
PLB	Phospholamban
SR	Sarcoplasmic reticulum
DADs	Delayed afterdepolarizations
INa	Fast sodium current
Ica	L-type calcium current
RAAS	Sympathetic–renin–angiotensin–aldosterone system
ERP	Effective refractory period
TNF-α	Tumor Necrosis Factor-alpha
IL-6	Interleukin-6
TGF-β1	Transforming Growth Factor-beta 1
MMP-9	Matrix Metalloproteinase-9
TG	Triglyceride
FBG	Fasting blood glucose
SD	Standard deviation
OR	Odds ratio
ACS	Acute coronary syndrome
NAFLD	Non-alcoholic fatty liver disease
HR	Hazard ratio
RFA	Radiofrequency ablation
AUC	Area Under the Curve
METS-IR	Metabolic Score for Insulin Resistance
TG/HDL-C Ratio	Triglyceride-to-HDL-Cholesterol Ratio
NOAF	New-onset AF
HOCM	Hypertrophic obstructive cardiomyopathy
ROC	Receiver Operating Characteristic
PCI	Percutaneous coronary intervention
STEMI	ST-segment elevation myocardial infarction
AMI	Acute myocardial infarction
OPCABG	Off-pump coronary artery bypass grafting
VAI	Visceral adiposity index
CABG	Coronary artery bypass grafting
MACCEs	Major cardiovascular and cerebrovascular events
MACEs	Major adverse cardiovascular events
ICU	Intensive care unit
